# The challenges of preventing drowning in Iran 

**Published:** 2022-01

**Authors:** Ali Davoudi Kiakalayeh, Mostafa Golshekan, Enayatollah Homaie Rad

**Affiliations:** ^*a*^ Guilan Road Trauma Research Center, Guilan University of Medical Sciences, Rasht, Iran.

## Abstract

**Background:**

Drowning is the second leading cause of death due to unintentional injury in northern Iran. Unlike other public injuries in Iran such as road traffic injury, only one drowning prevention program has been formally evaluated in this area of the country. The aim of this study is to describe the effect of the challenging program of drowning prevention on the reduction of drowning mortality cases in the north of Iran.

**Methods:**

We combined the data of the Iran National Registry of Drowning (INRD) and interviewed stockholders about the drowning prevention program to identify the challenges of drowning prevention in Iran. This type of research was thought to be the most appropriate for looking at events that could involve emotions. To access the opinion and perspective of the resident population in the rural settings of the study area regarding the drowning event, interviews were performed with the local elites including elected representatives, school teachers, religious leaders, and the victim’s families.

**Results:**

Based on drowning data, the registry was carried out by Guilan Road Trauma Research Center as a national center responsible for registering the drowning data through interviewing with lifeguards and health workers by the national focal point of drowning. Several factors have been identified as increasing the likelihood of drowning in northern Iran. The main factors included rip currents in the Caspian Sea, recirculation, buffer, standing waves, and Eddy line in rivers.

**Conclusions:**

This survey demonstrated that a prevention program for drowning can be sustainable when high-quality local drowning data are employed to target and model community-based injury prevention, and evaluate the outcomes. Local governments should install barriers for controlling access to water around rivers and canals and develop training programs on swimming, water safety, and safe rescue skills for the less than 18-year-old population by the Red Crescent Society.

**Keywords:**

Drowning, Prevention, INDR, Iran

